# Sex and Gender-Based Differences in Outcomes of Cardiac Rehabilitation Following Acute Myocardial Infarction

**DOI:** 10.1007/s11883-025-01360-5

**Published:** 2025-10-31

**Authors:** Alexis E. McFeely, Vera A. Bittner

**Affiliations:** 1 Heersink School of Medicine, University of Alabama at Birmingham, Birmingham, AL USA; 2 Division of Cardiovascular Disease, University of Alabama at Birmingham, GSB 444, 521 19th Street South, Birmingham, AL 35233 USA

**Keywords:** Cardiac rehabilitation, Acute myocardial infarction, Gender gap, Secondary prevention, Women-Focused cardiac rehabilitation

## Abstract

**Purpose of Review:**

This literature review summarizes recent interventions to enhance cardiac rehabilitation (CR) participation, enrollment, and completion among women and their impact on CR outcomes.

**Recent Finding:**

Despite well-documented benefits of CR, CR remains underutilized nationally and globally, more so among women than men. Barriers to CR participation reported by women have not changed significantly over time. Recent approaches to improving women’s enrollment, adherence, and completion have included home-based CR and telerehabilitation, incorporation of more diverse exercise modalities, and women-focused or women-only CR programming. Despite some encouraging results, most approaches have shown only modest benefits, and study interpretation is often limited by small study sizes, a lack of randomization, and highly selected samples.

**Summary:**

CR remains underutilized among women, contributing to poor health outcomes. Novel approaches to CR show promise, but further research is necessary to evaluate their impact on cardiovascular events, physiologic outcomes, and quality of life.

## Introduction

Cardiovascular disease (CVD) remains the national and global leading cause of death for women [1]. Much research has contributed to our current understanding of the sex-based differences in cardiac disease pathophysiology and presentation [2]. Nevertheless, women with CVD have historically been less likely to undergo revascularization or receive guideline-directed medical therapies, including cardiac rehabilitation (CR) [1, 3]. 

CR represents an individualized and multi-faceted approach to patient care designed to restore health-related quality of life (HRQoL), enhance exercise capacity, and reduce cardiac mortality [4]. According to Sect. 1861(eee)(4)(A)(ii) of the Social Security Act, CR is indicated in the US in patients with a history of acute coronary syndrome (ACS), percutaneous coronary intervention (PCI), coronary artery bypass grafting (CABG), stable chronic heart failure, heart transplant, and heart valve repair or replacement among several other cardiovascular conditions [5, 6]. In 2024, the American Association of Cardiovascular and Pulmonary Rehabilitation (AACVPR) and American Heart Association (AHA) updated the CR core components and addressed expanded CR delivery methods [4]. CR, in its present form, integrates a variety of services provided by a multidisciplinary care team, incorporating physician-prescribed aerobic and strength training exercises, as well as efforts to improve modifiable cardiovascular risk factors (e.g., dyslipidemia, hypertension, diabetes, and tobacco use) through education, counseling, and psychosocial management [4]. 

Over the past decade, many studies have investigated the effectiveness of novel delivery methods and CR components in enhancing health literacy, HRQoL, CR access, CR adherence, and overall health outcomes. Despite the established benefits of CR, current evidence suggests that less than a quarter of eligible Americans participate [7–10]. 

**Fig. 1:**
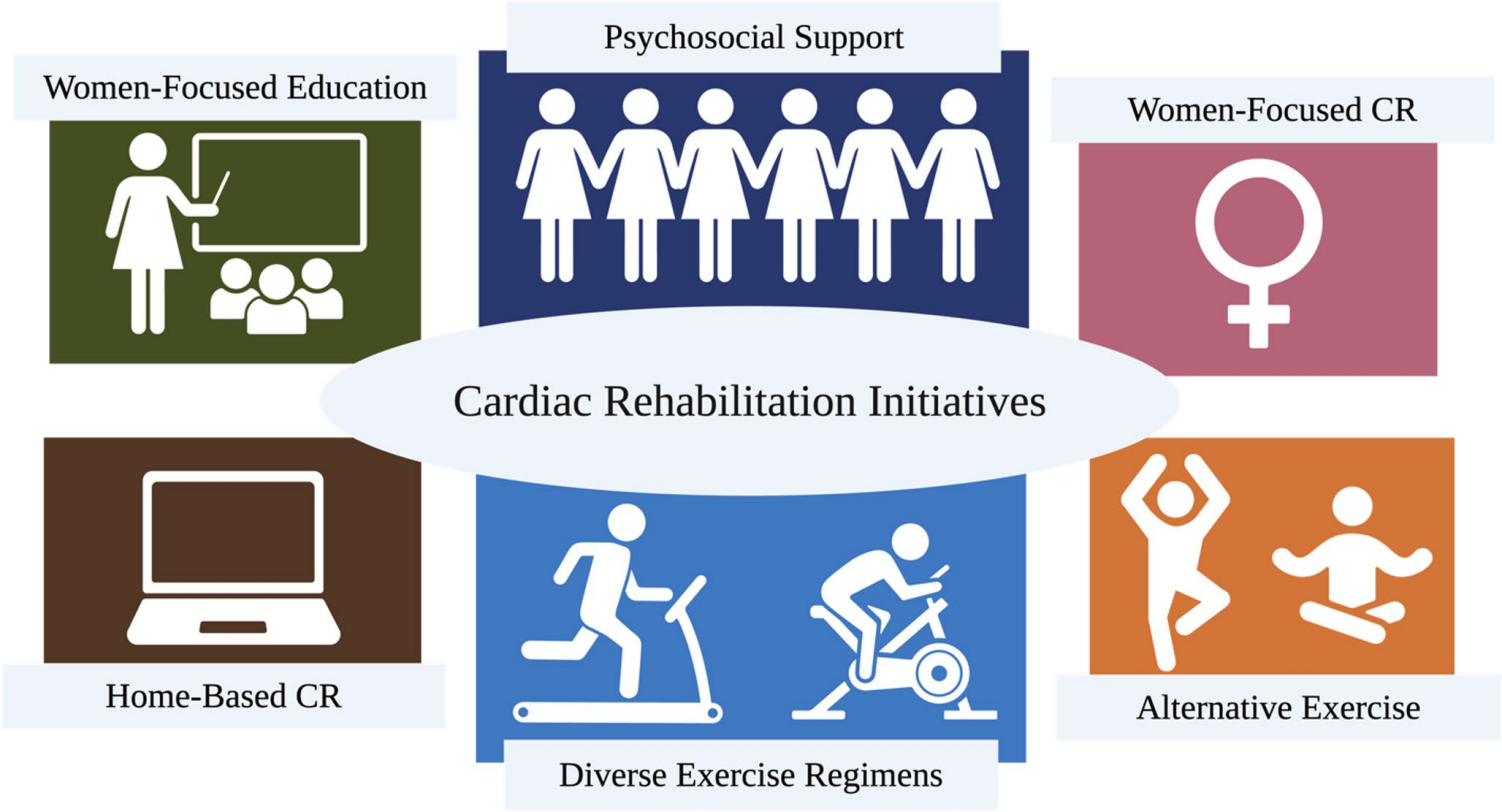
Several key cardiac rehabilitation interventions designed to enhance CR adherence and improve cardiovascular outcomes in women following ACS. *This figure was created using BioRender.com*

Women tend to present with ACS at an older average age and with a higher burden of comorbidities. Low utilization of CR among this population thus represents a significant concern [1, 3, 11, 12]. In the last ten years, there has been an increase in research to identify the unique needs of women and investigate potential interventions to reduce gender-based disparities in CR participation (Table 1) [13, 14]. In addition to the development and evaluation of women-focused CR programs (WFCR), recent studies have assessed the efficacy of cardiac telerehabilitation [15], women-centered educational programs [16], diverse forms of physical activity [17], and unconventional exercise regimens [18, 19] (Fig. 1). This paper is an update of a previous review published in 2018 [20]. 

## CR Outcomes among Women who Have Experienced ACS

Sex-specific differences in ACS outcomes have been well-documented nationally and globally, with research highlighting variations in pathophysiology, presentation, diagnostic timelines, and treatment [2, 3, 21, 22]. Recently, emphasis has been placed on better understanding these gender gaps, particularly concerning secondary CVD prevention. Bucholz et al. described young women (≤ 55 years) as a “distinct, higher-risk population.” [11] When compared with age-matched men, women were found to have a more significant burden of cardiovascular risk, including but not limited to factors such as diabetes and obesity [11]. In addition to an increased prevalence of comorbidities, women tended to present with MI later and with increased psychological distress (PHQ-9 ≥ 10) and reduced baseline QoL when compared with men [11]. Several studies have shown that women experience recurrent MACE after MI earlier than men and may have greater mortality within the first year [3, 12, 23]. CR participation may thus be particularly important among women.

Several recent studies highlight the benefits of CR on exercise capacity in women and men. Kasperowicz et al. assessed CR in post-ST-segment elevation myocardial infarction (STEMI) patients, identifying a statistically significant increase in exercise capacity in both genders upon completion of the program. Although men achieved numerically greater improvements, statistically significant between-group differences for 6-minute walk distance (6MWD) or MET scores were not observed [7]. Araya-Ramírez et al. similarly reported an increase in 6MWD for both genders after CR participation, with men experiencing greater increases in distance (429.3 ± 94.6 vs. 557.6 ± 90.7 m) compared with women (374.9 ± 100.7 vs. 483.2 ± 82.9 m), both *p* < 0.001 [24]. 

CR participation results in long-term improvements in modifiable risk factors. A gender-stratified analysis among post-MI CR patients showed statistically significant improvements in smoking cessation, triglycerides, and days of reported physical activity per week among men and women compared to the non-CR control [25]. Statistically significant differences in weight, BMI, and mean systolic blood pressure following CR were only observed in men, whereas significant differences in total cholesterol were detected exclusively in women [25]. 

In addition to decreasing recurrent MACE, CR has been shown to reduce cardiac mortality. In a meta-analysis by Ji et al., mortality (HR 0.47; 95% CI 0.56 to 0.39), risk of MACE (RR 0.49; 95% CI 0.44 to 0.55), and recurrent MI (RR 0.63; 95% CI 0.57 to 0.70) were reduced in CR participants compared to non-participants [8]. A 2023 update to the Cochrane Systematic Review by Dibben et al. similarly identified decreased cardiovascular mortality (RR 0.74; 95% CI 0.64 to 0.86), hospitalizations (RR 0.77, 95% CI 0.67 to 0.89), and MI (RR 0.82; 95% CI 0.70 to 0.96) [26]. Neither meta-analysis reported gender-specific outcomes, but Dibben et al. noted an increase in female representation from 11% to 17% over the past decade [26]. 

## Current Gender-Related Barriers to CR Utilization

A 2020 US study by Ritchey et al. among CR-eligible Medicare beneficiaries found 18.9% participation among women and 28.6% among men [9]. A 2023 international meta-analysis reported an average CR participation rate of 34% (95% CI 21% to 46%) in patients with MI, with men more likely to participate than women (Odds Ratio (OR) 1.690; 95% CI 1.276 to 2.239), *p* < 0.001 [27]. 

A 2017 systematic review by Supervia et al. identified 25 studies that had explored barriers to CR participation among women [14]. Key patient-level barriers included older age, comorbidities, and lack of information or negative beliefs about CR [14]. Social environmental obstacles included a lack of referral, transportation problems, family obligations, financial concerns, inadequate insurance, and a lack of social support from friends and family [14]. Strong endorsement by a healthcare professional correlated with increased CR participation [14]. A small qualitative study among 10 women following CR dropout identified mental health issues, scheduling conflicts with caretaking obligations, lack of transportation, and an incomplete understanding of CR benefits as central barriers to program adherence [28]. 

In a 2023 international, cross-sectional study of CR underutilization, Ghisi et al. highlighted a similar constellation of barriers to female participation, including lack of knowledge about CR, failure of the CR program to reach out following referral, expense, and a negative perception of exercise [29]. Program adherence was adversely affected by distance from the CR site, travel, family responsibilities, and difficulties in accessing in-person sessions [29]. Although specific challenges varied between men and women, sex-based differences in total CR barriers as assessed by the Cardiac Rehabilitation Barriers Scale (CRBS) did not differ by gender but showed substantial regional variation [29]. These results suggest that region-specific assessment and interventions will be required to improve CR attendance among women and men [29]. Using the Persian model of the CRBS, a 2023 Iranian cross-sectional study of 1,053 cardiac patients showed higher average CRBS scores in women (2.37 ± 0.37) than men (2.29 ± 0.35) [30]. The study highlighted the disproportionate impact of distance to the program, cost, and limited access to transportation, as well as a high burden of comorbidities among women [30]. 

A 2024 scoping review of 10 studies from 6 countries reported that women had lower rates of CR participation than men following MI [31]. Increased “caring responsibilities” and physician referral bias were identified as potential barriers to female enrollment and adherence, while a high burden of comorbidities, lower socioeconomic status, employment situation, and poor awareness of the value of CR limited access for both genders [31]. Moreno et al. evaluated patients after MI at 6 and 12 months to explore factors associated with failure to adhere to secondary prevention recommendations [32]. Caregiver burden (adjusted OR, 1.45; 95% CI 1.08 to 1.94) and depression (adjusted OR, 1.40; 95% CI 1.03 to 1.92) were more common in women than men and negatively associated with adherence to preventive measures [32]. 

Patient dissatisfaction can lead to poor CR adherence. A 2017 study of CR participants measured satisfaction at intake, discharge, and 6 months, 1 year, and 2 years following CR completion [33]. Women had higher satisfaction scores than men (3.02 ± 1.12 vs. 2.66 ± 1.14). CR adherence and satisfaction were positively correlated (*r* = 0.22, *p* < 0.01) [33]. Future studies should investigate whether improving programs and enhancing patient satisfaction leads to increased CR retention rates. Nonetheless, high satisfaction among women enrolled in CR suggests that reliable referral pathways to enhance enrollment may be an effective strategy for improving utilization.

**Table 1:** Recent key studies exploring CR outcomes and Gender-Related barriers to CR

Author (Year)	Country		Study Details	Proportion of Women	Summary of Major Findings	
Kasperowicz et al. (2019) [7]	Poland	Retrospective chart review of 100 STEMI patients undergoing CR		40%	• Exercise capacity improved among participants. • Positive changes were noted among men and women (6MWD and METs).	
Araya-Ramírez et al. (2022) [24]	Costa Rica	Retrospective chart review of 311 patients enrolled in a 12-week CR program		~ 24%	• Men and women experienced an increase in mean 6MWD. • A decreased diastolic blood pressure was observed among both groups.	
Ji et al. (2019) [8]	International	Meta-analysis of 25 studies (55,035 ACS patients) published between 2010 and 2018 on PubMed, Web of Science, or EMBASE		Not reported	• A significant reduction in the risk of cardiac death and incidence of MACE was observed among CR participants. • No gender-stratified analysis	
Dibben et al. (2023) [26]	International	Meta-analysis of 85 RCTs (23,430 CAD patients) published between June 2014 and September 2020 on CENTRAL, MEDLINE, EMBASE, CINAHL, SCIE, ICTRP, and ClinicalTrials.gov		< 20%	• Reductions in risk across three outcome measures were observed among CR participants: (1) cardiovascular mortality, (2) hospitalizations, and (3) MI. • HRQoL improved among participants, with 63% of studies reporting an increase in at least one subscale. • No gender-stratified analysis	
Sjölin et al. (2020) [25]	Sweden	Retrospective registry-based cohort study of 19,136 post-MI patients in the Swedish heart disease registry (SWEDEHEART)		25%	• Men and women enrolled in CR had higher rates of smoking cessation and increased activity than non-participants at 1 year. • Women experienced significant reductions in total cholesterol and low-density lipoprotein cholesterol.	
Ritchey et al. (2020) [9]	United States	Retrospective observational cohort study of 366,103 CR-eligible beneficiaries		~ 43%	• CR participation was lower among women than men. • Only a quarter of CR-eligible patients participated in CR, of whom less than one third completed ≥ 36 sessions.	
Wang et al. (2023) [27]	International	Global meta-analysis including 14 studies (114,542 MI patients) from ten databases		Not Reported	• CR participation rates were low (34%) among MI patients. • Women were less likely than men to utilize CR after AMI.	
Ghisi et al. (2023) [29]	International	Cross-sectional survey of 2,163 patients from 16 countries conducted between 2021 and 2023		~ 42%	• Barriers to CR were reported to differ greatly based on region. • Significant barriers to CR enrollment and adherence were identified.	
Moreno et al. (2024) [32]	Spain	Prospective observational cohort study of 503 MI patients exploring secondary prevention adherence		~ 20%	• Caregiver burden and depression (two independent predictors of global non-adherence to key secondary prevention measures following MI including Mediterranean diet, physical activity, and CR) were identified more commonly in women.	

## Interventions: Strategies to Enhance CR Efficacy and Reduce Gender Gap

The literature published before 2018 highlighted a variety of interventions designed to improve CR utilization among women, including automated or liaison referral to counteract provider bias, participation incentives, individualized coaching, and women-only CR groups, among others; however, data to support their efficacy and feasibility were limited [20]. Though home-based CR (HBCR) and technology-based interventions (e.g., telemedicine and a web-based approach) had been proposed at that time, there was insufficient data to support their implementation [20]. In recent years, strides have been made to further evaluate many of these approaches and develop novel initiatives that enhance CR utilization across genders, with several approaches specifically focused on women (Table 2).

### Home-Based Cardiac Rehabilitation (HBCR) and Telemedicine

CR disruptions related to the 2020 COVID-19 pandemic were a driving force behind the push toward alternative CR strategies [34, 35]. In 2021, Ghisi et al. reported surveys of 1,062 CR programs across 70 countries and found that almost half of all responding programs had ceased entirely, and 27.1% reported temporary suspension, averaging 8.3 ± 2.8 weeks [35]. Telerehabilitation emerged as a particularly promising alternate CR delivery approach [34, 36–38]. 

Recent literature appears to uphold technology-enabled CR approaches, such as HBCR and hybrid CR programs, as effective alternatives to center-based CR (CBCR) for both men and women [39, 40]. Similar to CBCR, HBCR may enhance cardiovascular health, satisfaction, and exercise endurance, while reducing all-cause mortality and unplanned hospitalizations [41–44]. AACVPR, AHA, and ACC released a statement in 2019 cautiously supporting the use of HBCR in “low- to moderate-risk patients who are eligible for CR but cannot attend a traditional center-based CR program” [45]. The authors examine the benefits, drawbacks, and core components of HBCR, characterizing it as a promising alternative while highlighting the need for further research [45]. Though a multitude of recent studies have investigated the utility of telemedicine-supported CR programs, few have specifically evaluated their impact on female enrollment, adherence, and health outcomes.

A 2025 randomized controlled trial (RCT) compared the outcomes of mobile health CR (mHealth-CR) with traditional CBCR through evaluation of 6MWD among 400 participants aged 65 and older (30% women) [46]. There was no clinically significant (≥ 25 m) difference in 6MWD between experimental groups overall (15.6 m; 95% CI − 0.3 to 31.5 m), but an exploratory analysis stratified by gender identified a significant increase among women (36.6 m; 95% CI 8.7 to 64.4 m) [46]. This finding opens up the intriguing possibility that mHealth-CR may be particularly suitable for women [46]. A 2023 cross-sectional study investigating the effects of HBCR among patients with ischemic heart disease reported that women experienced more substantial reductions in anxiety than men (−25 ± 0.4 vs. −15 ± 1.1; *p* = 0.03), but limited female representation (15%) reduces the reliability of the results [15]. In contrast to the above studies, a 2024 prospective cohort study by Hou et al. reported female gender as a negative predictor of HBCR participation and cardiovascular outcomes [47]. The study did not report baseline comorbidities or fitness by gender, and a lower proportion of women participated in the HBCR group (18.5%) when compared with the usual care group (32%). Both factors may have contributed to the findings.

Few women-only studies have explored HBCR. Zhou et al. compared the efficacy of low-frequency HBCR and high-frequency CBCR among middle-aged women following MI and coronary stenting using a case-control design [44]. Oxygen consumption, anaerobic threshold, exercise duration, and QoL increased significantly in both groups, but only CBCR showed statistically significant improvements in BMI, body composition, blood glucose, and high-density lipoprotein cholesterol. Lipid profiles improved in both groups, but a high rate of lipid-lowering medication use confounds the results [44]. A 2023 women-only longitudinal cohort study compared HBCR and CBCR and found only minimal differences in all-cause hospitalizations (HBCR 17%, CBCR 18.1%, OR 1.14; 95% CI 0.66 to 1.96) [48]. Women who chose to participate in HBCR tended to live farther away from healthcare facilities than those enrolled in CBCR and completed a greater number of total sessions, suggesting that technology-enabled HBCR may reduce barriers to enrollment and adherence [48]. 

A 2025 RCT evaluated the impact of a novel Technology-based Cardiac Rehabilitation Therapy (TaCT) program on functional capacity, management of cardiovascular risk factors, and well-being among women with heart disease [49]. The smartphone-based program involved an educational website and app, guided yoga and mindfulness videos, and biweekly phone-based sessions [49]. When compared to the routine care group, TaCT yielded significant improvements in functional capacity, QoL, participation in cardioprotective behaviors, and reductions in anxiety (*p* < 0.05) [49]. Menezes et al. did not observe intergroup differences in blood pressure, cardiac symptoms, or body composition measures [49]. These findings highlight the potential of TaCT and similar virtual models to enhance health outcomes among women, particularly in middle- to low-income settings.

Telehealth-enabled CR approaches appear to enhance adherence, potentially addressing significant barriers to participation, including distance, transportation, and scheduling difficulties, and showing encouraging physiologic changes in response to the intervention [29]. Despite this, the majority of studies evaluating HBCR fail to include sufficient numbers of female participants and therefore lack the power to conduct gender-stratified analysis. Further research on the utility of CBCR vs. HBCR in women is needed.

### Diverse Exercise Regimens

CR has traditionally utilized moderate-intensity continuous training (MICT). However, the past decade has seen a growing interest in evaluating the efficacy of alternative exercise strategies in CR, including high-intensity interval training (HIIT). With an increasing body of evidence to support its effectiveness, further investigation and eventual incorporation into CR programs may be warranted [50–52]. 

A 2025 RCT involving 100 post-MI patients compared the impact of MICT and HIIT on cardiac function and found that both methods were effective in improving echocardiographic indices of myocardial function [53]. Gonçalves et al. compared the impact of HIIT vs. MICT on blood pressure, body composition, and several biomarkers, noting statistically significant improvements among both HIIT and MICT patients when compared with a no-intervention control group [54]. HIIT was more effective than MICT in improving body composition, lipid profile, and hemoglobin A1c in addition to several other cardiovascular biomarkers [54]. 

HIIT has also been reported to improve health outcomes among women specifically. A 2021 RCT involving 56 women compared clinical outcomes following CR participation in either MICT with moderate-intensity resistance training (RT) or HIIT combined with a more rigorous RT regimen [55]. Patients in the HIIT intervention group demonstrated significantly larger increases in leg strength and absolute peak VO_2_ than MICT control patients (peak VO_2_ + 23% and + 7%, respectively), suggesting that a combined HIIT-RT could enhance outcomes among women enrolled in CR [55]. A study assessing the effectiveness of combined HIIT and MICT vs. MICT only as a means of improving cardiovascular endurance in post-menopausal women with CAD also found greater increases in peak VO_2_ among HIIT participants (+ 0.95 mL kg^−1^ min^−1^; *p* < 0.001), but noted difficulties achieving and maintaining the target heart rate at 90%−95% of peak as well as potential barriers such as joint pain and comorbidities [19]. 

Reed et al. explored the effects of a long-interval HIIT protocol compared to MICT on physical and mental health in a case-control study of female CR participants [56]. They reported statistically significant reductions in depression (− 0.7 ± 3.0 vs. −1.1 ± 2.3, *p* = 0.003) and anxiety scores (− 1.8 ± 3.5 vs. −1.5 ± 3.0, *p* < 0.001) in the HIIT group as well as a favorable impact on BMI, waist circumference, and resting diastolic blood pressure [56]. In a small qualitative group interview study, Lee et al. highlighted emotional barriers to interval training, such as anxiety associated with performing intense exercise and challenges due to comorbid conditions [57]. However, participants also reported feeling “a sense of accomplishment and purpose” [57]. 

HIIT may not be suitable for all women in CR, but selected women may derive significant benefit compared to MICT. Larger studies with diverse groups of patients will need to be conducted to determine the safety and efficacy of HIIT among women in CR.

### Alternative Forms of Exercise

Over the past decade, researchers have proposed the implementation of alternative exercise in CR, suggesting that such methods may enhance enrollment and adherence among women [58]. A diversity of exercise modalities (e.g., dance, sports, Tai Chi, Pilates, group walking, Nordic walking, and yoga, among others) may provide women with a more engaging and motivating CR experience, while improving exercise endurance, QoL, and enjoyment [58–61]. 

The most extensive study to date is the Yoga-CaRe trial published in 2020. This RCT involved 3,959 patients across 24 healthcare facilities in India and compared MACE and self-rated health between participants in a yoga-based CR and an enhanced standard care group [17]. There was no meaningful difference in rates of MACE between groups. Self-health scores adjusted for baseline were higher in the yoga group (1.5; 95% CI 0.5 to 2.5; *p* = 0.002), and participants reported greater return to pre-infarct activities at 12 weeks [17]. The researchers reported no sex-based difference in CR adherence following enrollment, but the proportion of female participants was only 14% [17]. A small Australian pilot study of a Women’s Yoga CR Program (WYCRP) showed higher rates of completion (95% vs. 56%) and adherence (72% vs. 12%) than traditional CR [62]. Moreover, qualitative data from focus group sessions with WYCRP patients indicated that yoga-based CR may reduce anxiety associated with physical activity, enhance mood, and increase self-confidence [62]. Despite promising data, evidence to support the use of yoga and other forms of alternative exercise remains limited. A 2025 meta-analysis by Suebkinorn et al. specifically underscored the need for women-focused RCTs to more accurately assess the effects of these interventions on CR completion rates, exercise capacity, QoL, and other health outcomes [59]. 

### Women-Focused CR (WFCR)

Recent studies have discussed the feasibility, advantages, and potential challenges of programs designed to “better engage women” and to more effectively address their unique post-MI cardiovascular risk profile [63, 64]. In 2022, the first guidelines for WFCR were developed by the International Council of Cardiovascular Prevention and Rehabilitation (ICCPR), featuring 15 evidence-based recommendations regarding referral, setting, and delivery models [63]. A 2024 international CR program survey found that less than 15% of responding programs offered female-centered components [65]. The most frequently cited barriers to WFCR delivery were a lack of physical resources, insufficient time, inadequate space, a shortage of human and educational resources, and limited staff expertise [65]. 

In a 2021 systematic review, only a third of WFCR programs incorporated gender-tailored content, and less than 20% employed alternative exercise modalities [66]. In a later 2022 meta-analysis of 28 studies, Mamataz et al. explored outcomes of WFCR. Relative to the active comparison group (traditional or home-based CR), WFCR programs did not produce increases in METs or peak VO_2_ [67]. However, they were associated with enhanced QoL as demonstrated by increases in SF-36 scores in 7 of the 8 domains [67]. The quality of evidence is moderate-to-low, highlighting the need for larger-scale trials [67]. 

#### Women-Only CR

Women-only CR is a component of the WFCR approach that has been investigated to a limited extent over the past decade. Women-only CR availability remains low worldwide, but with significant variability between countries and greater availability in high-resourced programs [68]. The efficacy of women-only CR is unclear. A 2021 retrospective study by Heald et al. found less favorable outcomes in a non-gender-tailored women-only CR program compared to mixed-sex CR, with both groups of women improving their cardiovascular risk factors to a lesser degree than a matched group of male CR participants [64]. A later study by the same research team found that women may not necessarily prefer women-only CR [69]. Only 22% of women opted to participate in women-only CR compared to 74.7% who chose mixed-sex CR, and 3.3% who chose home-based CR [69]. Mixed-sex CR participants also demonstrated higher rates of CR adherence to the 25 sessions offered compared to women-only CR participants (18.2 ± 5.3 vs. 16.6 ± 4.6; *p* < 0.001) [69]. It is unknown whether the implementation of gender-tailored components would have yielded better results. Together with the findings of studies assessing comprehensive, guideline-directed WFCR programs, recent literature suggests that program content, rather than participant composition, may be responsible for the positive outcomes.

#### Psychosocial Support

Psychosocial support during CR is essential for both genders. Despite research suggesting gender-specific variability in psychological outcomes after MI [70, 71], studies assessing specific psychosocial interventions among women have been limited over the past decade. Though psychosocial counseling and other forms of support have been offered as components of WFCR in a variety of studies [66], there is no recent research to specifically link such support to CR outcomes in women.

#### Female-Centered Education

Education is a core component of CR. The *Cardiac College for Women* (https://www.healtheuniversity.ca/en/cardiaccollege) is a female-focused educational CR curriculum consisting of 12 main sessions covering an array of topics (hypertension, menopause, medication adherence, mindful eating, psychosocial health, and post-program maintenance, among others) designed to enhance female health literacy and outcomes [72]. The program webpage also presents information on other cardiovascular diseases that disproportionately affect women (e.g., Takotsubo cardiomyopathy, SCAD, MINOCA, polycystic ovarian syndrome, and postmenopausal hormone therapy) [72]. Several studies have assessed the efficacy of this educational initiative. A 2024 multi-site mixed-method study among 40 post-CR women reported an 80% attendance rate across the 12 sessions, and the curriculum was well-received [73]. Ghisi et al. in a 2025 multi-site pilot study suggested that women-focused health education yielded improvements in cardiovascular health knowledge, QoL, functional capacity, and compliance with a Mediterranean diet [16]. When compared to standard co-educational CR patients, the *Cardiac College* participants experienced statistically significant improvements in cardiac knowledge (*p* < 0.05) and dietary compliance (*p* = 0.04) [16]. Large-scale randomized studies are needed to confirm these results.

**Table 2:** Recent key studies exploring CR interventions to reduce the gender gap

Author (Year)	Country	Study Details	Proportion of Women	Summary of Major Findings
Dodson et al. (2025) [46]	United States	Multi-center RCT of 400 patients with ischemic heart disease across 5 university hospitals comparing the effects of mHealth-CR and usual care on 6MWD	27.2%	• Changes in 6MWD did not differ significantly between groups. • Subgroup analysis identified a clinically significant (> 25 m) improvement in 6MWD between mHealth-CR and usual care.
Calvo-Lopez et al. (2023) [15]	Spain	Prospective study of 62 post-MI patients involved in HBCR	15%	• Cardiac telerehabilitation resulted in improvements in physical and emotional health. • Women had higher levels of preintervention anxiety but greater postintervention reductions than men.
Hou et al. (2024) [47]	China	Prospective cohort study of 1,533 post-MI patients comparing HBCR with CBCR	~ 29.5%	• Female gender was identified as a negative predictor of HBCR participation and effectiveness.
Zhou et al. (2023) [44]	China	Case-control study involving 90 women following stenting, comparing low-frequency HBCR to high-frequency CBCR	100%	• High-frequency CBCR was more effective at improving certain biomarkers than low-frequency HBCR. • Both designs improved risk factors for cardiovascular disease, fitness, and QoL.
Najem et al. (2023) [48]	United States	Longitudinal cohort study of 753 women participating in either HBCR or CBCR	100%	• Technology-enabled HBCR and CBCR have comparable 12-month hospitalization rates. • HBCR patients lived at greater distances from the medical center and attended more sessions than those in CBCR.
Menezes et al. (2025) [49]	India	Single-center, single-blind superiority RCT of 100 women comparing TaCT vs. standard care CR in a middle-income region	100%	• Compared with standard care, TaCT significantly improved functional capacity, cardioprotective behaviors, QoL, and anxiety among women. • TaCT may enhance the health outcomes of women in middle-income areas.
Obert et al. (2025) [53]	France	RCT comparing the effects of HIIT vs. MICT on myocardial function in 100 post-MI patients	10%	• HIIT and MICT both effectively enhanced cardiorespiratory fitness and cardiac function while reducing cardiac risk. • HIIT improves cardiovascular health outcomes in post-MI patients.
Gonçalves et al. (2024) [54]	Portugal	RCT of 72 patients with CAD comparing HIIT, MICT, and control CR	14%	• HIIT and traditional MICT both yielded beneficial cardiovascular outcomes. • HIIT produced larger cardiovascular and metabolic benefits than MICT (e.g., systolic blood pressure, body fat mass, waist circumference, and biomarkers).
Khadanga et al. (2022) [55]	United States	RCT comparing HIIT combined with intensive RT vs. standard MICT with moderate-intensity RT	100%	• HIIT + RT was associated with a greater increase in leg strength and mean peak VO_2_ when compared with the control.
Lee et al. (2019) [19]	Canada	RCT investigating the effects of HIIT + MICT vs. MICT only on exercise capacity among 31 women with CAD	100%	• Though high attrition rates in both groups compromised the study design, improvement in peak VO_2_ was significantly greater among the HIIT group when compared with MICT (*p* < 0.005).
Reed et al. (2019) [56]	Canada	RCT comparing the effects of long-interval HIIT vs. MICT on psychological and physical health outcomes in 60 women with CVD	100%	• Compared to MICT, HIIT produced greater improvement in anxiety and depression. • Improvements in clinical measures of cardiometabolic health were identified in both groups.
Lee et al. (2022) [57]	Canada	Qualitative, group interview study of 9 women with CAD who participated in the 2019 Lee et al. study	100%	• Deductive thematic analysis found that HIIT produced anxiety and fear of discomfort in some women, while engendering enthusiasm, excitement, pride, and accomplishment in others. • Patient experience with HIIT may depend on comorbidity burden.
Prabhakaran et al. (2020) [17]	India	RCT of 3,959 post-MI patients from 24 centers comparing Yoga-CaRe vs. enhanced standard care	~ 14%	• Compared to the control, the Yoga CaRe program improved self-rated health (77.0 ± 16.8 vs. 75.7 ± 17.8; *p* = 0.002) and return to pre-MI activities (88.3 ± 18.9 vs. 87.0 ± 20.1; *p* = 0.039). • There was no gender-based difference in adherence to Yoga-CaRe.
Murphy et al. (2021) [62]	Australia	Pilot study of 27 CR-eligible women assessing the Women’s Yoga CR Programme (WYCRP) compared to usual CR	100%	• 81% of patients opted to participate in WYCRP. • Though no significant difference was found in attendance, completion (95% vs. 56%; *p* = 0.02) and program adherence (72% vs. 12%; *p* < 0.001) were greater in the WYCRP group.
Suebkinorn et al. (2025) [59]	International	Meta-analysis of 8 RCTs involving 398 women from 9 databases assessing alternative exercise in CR	100% (≥ 50% in RTCs)	• Despite small short-term benefits, alternative exercise had little effect on CR completion and several health outcomes compared to traditional CR. • Evidence is limited due partly to the underrepresentation of women in most trials.
Ghisi et al. (2024) [65]	Global (6 WHO regions)	Cross-sectional, survey-based study of 223 CR programs across 52 countries	Not Applicable	• 14.8% of programs offered some form of WFCR, though many failed to offer key guideline-directed, gender-tailored components.
Mamataz et al. (2022) [67]	Multinational	Meta-analysis of 28 studies (*n* = 3,697 women with heart disease) identified from a search of 8 databases	100%	• Though WFCR did not produce significant improvement in functional capacity when compared to other active forms of CR, it yielded improvements in mental and physical QoL.
Turk-Adawi et al. (2021) [68]	Global	Cross-sectional survey study of 1,082 CR programs across 93 countries to assess global women-only CR delivery	Not Applicable	• Only 40.9% of countries and 11.8% of surveyed CR programs offered women-only CR. • Limited access to resources was identified as a potential barrier to women-only CR delivery.
Heald et al. (2021) [64]	Canada	Retrospective study of 727 women participating in mixed CR, women-only CR, or HBCR	~ 61.6%	• Patients in mixed-sex CR experienced a greater peak VO_2_ than non-gender-tailored women-only CR participants. • Sex-based disparities in cardiovascular risk existed pre-/post-intervention.
Heald et al. (2022) [69]	Canada	Retrospective cohort study of the 727 participants in the previous 2021 study	100%	• Adherence was higher in the mixed-sex group compared to women-only group (58.8 ± 28.9% sessions/25 vs. 54.3 ± 26.3% sessions/25; *p* = 0.046), but completion rates of both programs were higher than those seen in HBCR.
Carson et al. (2024) [73]	Canada	Multi-site, mixed-method study of 40 women completing the *Cardiac College for Women* curriculum and 5 staff members at 2 Canadian CR programs	100%	• Attendance rates were high (80% of sessions). • Qualitative data demonstrated a generally positive female perception of the *Cardiac College for Women* curriculum.
Ghisi et al. (2025) [16]	Canada	Multi-site, prospective, pilot study of 62 women choosing to enroll in women-only CR or usual CR at 2 Canadian centers	100%	• Completion of the *Cardiac College for Women* curriculum produced significant improvement in cardiac knowledge and dietary compliance when compared to standard CR (*p* < 0.05).

## Conclusions

This updated review highlights the persistent gender-based disparities in CR utilization and outcomes. While recent studies demonstrate the capacity of various gender-tailored interventions to enhance CR enrollment, adherence, and completion among women, further research is needed to quantify the physical and psychological benefits of these initiatives to address barriers to implementing these women-focused interventions.

## Key References

- Araya-Ramirez F, Moncada-Jimenez J, Grandjean PW, Franklin BA. Improved Walk Test Performance and Blood Pressure Responses in Men and Women Completing Cardiac Rehabilitation: Implications Regarding Exercise Trainability. American Journal of Lifestyle Medicine. 2022;16(6):772–8. 10.1177/1559827621995129.
  - This study evaluated changes in exercise capacity and blood pressure among men and women following participation in CR.
- Wang L, Liu J, Fang H, Wang X. Factors associated with participation in cardiac rehabilitation in patients with acute myocardial infarction: A systematic review and meta-analysis. Clinical Cardiology. 2023;46(11):1450–7. 10.1002/clc.24130.
  - This global meta-analysis explored CR participation rates among men and women following MI.
- Ghisi GLM, Kim WS, Cha S, Aljehani R, Cruz MMA, Vanderlei LCM, et al. Women’s Cardiac Rehabilitation Barriers: Results of the International Council of Cardiovascular Prevention and Rehabilitation’s First Global Assessment. Canadian Journal of Cardiology 2023;39(11 S): S375–S83. 10.1016/j.cjca.2023.07.016.
  - This international cross-sectional survey identified gender-specific differences in barriers to CR enrollment and adherence.
- Dodson JA, Adhikari S, Schoenthaler A, Hochman JS, Sweeney G, George B, et al. Rehabilitation at Home Using Mobile Health for Older Adults Hospitalized for Ischemic Heart Disease: The RESILIENT Randomized Clinical Trial. JAMA Network Open. 2025;8(1):e2453499. 10.1001/jamanetworkopen.2024.53499.
  - This RCT compared the effects of a mobile health CR (mHealth CR) program and standard CR on functional capacity among older adults, with key results stratified by gender.
- Khadanga S, Savage PD, Pecha A, Rengo J, Ades PA. Optimizing Training Response for Women in Cardiac Rehabilitation: A Randomized Clinical Trial. JAMA Cardiology. 2022;7(2):215–8. 10.1001/jamacardio.2021.4822.
  - This study evaluated the impact of a HIIT-centered training regimen vs. a MICT-based approach on changes in peak VO_2_ and leg strength among women.
- Prabhakaran D, Chandrasekaran AM, Singh K, Mohan B, Chattopadhyay K, Chadha DS, et al. Yoga-Based Cardiac Rehabilitation After Acute Myocardial Infarction: A Randomized Trial. Journal of the American College of Cardiology. 2020;75(13):1551–61. 10.1016/j.jacc.2020.01.050.
  - This study is the largest to date to explore yoga as an alternative to MICT in CR, highlighting its impact on self-rated health and return to baseline activity levels.
- Ghisi GLM, Supervia M, Turk-Adawi K, Beleigoli A, Contractor A, Mampuya WM, et al. Women-Focused Cardiac Rehabilitation Delivery Around the World and Program Enablers to Support Broader Implementation. CJC Open. 2024;6(2Part B):425–35. 10.1016/j.cjco.2023.10.008.
  - This study examines the global prevalence of WFCR programs while identifying common barriers to their delivery.
- Mamataz T, Ghisi GL, Pakosh M, Grace SL. Outcomes and cost of women-focused cardiac rehabilitation: A systematic review and meta-analysis. Maturitas. 2022;160:32–60. 10.1016/j.maturitas.2022.01.008.
  - This multinational study investigates the clinical benefits of WFCR, focusing on its impact on physical and mental QoL.
- Ghisi GLM, Hebert AA, Oh P, Colella T, Aultman C, Carvalho C, et al. Evidence-informed development of women-focused cardiac rehabilitation education. Heart & Lung. 2024;64:14–23. 10.1016/j.hrtlng.2023.11.004.
  - This study outlines the development of the Cardiac College for Women.

## Data Availability

No datasets were generated or analysed during the current study.

## Abbreviations

AACVPR: American Association of Cardiovascular and Pulmonary Rehabilitation
ACC: American College of Cardiology
ACS: Acute Coronary Syndrome
AHA: American Heart Association
BMI: Body Mass Index
CBCR: Center-Based Cardiac Rehabilitation
CR: Cardiac Rehabilitation
CRBS: Cardiac Rehabilitation Barriers Scale
CVD: Cardiovascular Disease
HBCR: Home-Based Cardiac Rehabilitation
HIIT: High-Intensity Interval Training
HRQoL: Health-Related Quality of Life
ICCPR: International Council of Cardiovascular Prevention and Rehabilitation
MACE: Major Adverse Cardiovascular Events
mHealth-CR: Mobile Health CR
MI: Myocardial Infarction
MICT: Moderate Intensity Continuous Training
QoL: Quality of Life
RCT: Randomized Controlled Trial
RT: Resistance Training
STEMI: ST-Segment Elevation MI
WFCR: Women-Focused Cardiac Rehabilitation
Yoga-CaRe: Yoga-Based CR Program
